# Evaluation of the Potency of Two Pyolysin-Derived Recombinant Proteins as Vaccine Candidates of *Trueperella Pyogenes* in a Mouse Model: Pyolysin Oligomerization and Structural Change Affect the Efficacy of Pyolysin-Based Vaccines

**DOI:** 10.3390/vaccines8010079

**Published:** 2020-02-10

**Authors:** Lingxiao Yang, Hongmin Liang, Bing Wang, Bo Ma, Junwei Wang, Wenlong Zhang

**Affiliations:** 1 Heilongjiang Key Laboratory for Animal Disease Control and Pharmaceutical Development, College of Veterinary Medicine, Northeast Agricultural University, Harbin 150030, China; yanglx0218@sina.com (L.Y.); lhm19950720@163.com (H.L.); wb199473@163.com (B.W.); mabo99@neau.edu.cn (B.M.); jwwang@neau.edu.cn (J.W.); 2 Northeastern Science Inspection Station, China Ministry of Agriculture Key Laboratory of Animal Pathogen Biology, Harbin 150030, China

**Keywords:** *Trueperella pyogenes*, pyolysin, recombinant antigens, vaccine efficacy, oligomerization and structural changes

## Abstract

*Trueperella pyogenes* (*T. pyogenes*) is an important opportunistic pathogen in livestock and wild animals. However, only one commercial *T. pyogenes* vaccine is currently available, and its immunoprotective effect is not ideal. Pyolysin (PLO) is one of the important virulence factors expressed by *T. pyogenes* and one of the targets for the development of new *T. pyogenes* vaccines. In this study, we constructed two recombinant antigens, tPLOA1 (contains amino acids 1–110 and domain 4 of the PLO molecule) and tPLOA2 (contains amino acids 190–296 and domain 4 of the PLO molecule). Vaccines were prepared by mixing the two recombinant antigens with incomplete Freund’s adjuvant or sheep red blood cell membrane and provided partial immune protection to immunized mice against the lethal challenge of *T. pyogenes.* Analysis of the PLO-specific IgG levels of immunized mice indicated that the antibody-inducing potency and immunoprotective efficacy of PLO-based vaccines are affected by the oligomerization and structural changes of PLO after binding to a cholesterol-containing membrane. In addition, the titer of anti-hemolysis antibodies is not a suitable indicator of the immunoprotective effect of these vaccines in PLO-based vaccine-immunized animals. The results provide new insights into the development of *T. pyogenes* vaccines.

## 1. Introduction

*Trueperella pyogenes* (*T. pyogenes*) is an important opportunistic pathogen in livestock and wild animals, and its infection generally leads to clinical diseases, such as metritis, mastitis, pneumonia, and liver abscess, in economically important animals, such as pigs, cattle, and sheep [1]. Antibiotics, such as tylosin and tetracycline, are used to prevent liver abscess caused by bacteria such as *T. pyogenes* [2]. However, the emergence of drug-resistant bacterial strains restricted the use of antibiotics in food-producing animals [3,4,5]. Therefore, alternative methods for the prevention of *T. pyogenes* infections are still needed. Vaccines are important for preventing and controlling infectious diseases. *T. pyogenes* vaccines have been developed since the 1950s [2]. *T. pyogenes* bacterin toxoid and genetic toxoid were widely tested for their potency against *T. pyogenes* infections or challenge in experimental animals and livestock [6,7,8,9,10]. Inactivated whole *T. pyogenes* culture was also used as vaccine antigen [11,12]. Inactivated *T. pyogenes* whole cells alone or combined with heterologously expressed bacterin were employed to formulate vaccines [13,14]. DNA vaccines were also prepared against *T. pyogenes* infection [15,16]. However, only one vaccine against *T. pyogenes* is commercially available at present, and its immune protective effect is not satisfactory based on a meeting report of the World Organization for Animal Health (OIE) ad hoc group, which discussed the prioritization of diseases and determined the vaccines that could reduce antimicrobial use in cattle, sheep, and goats.

*T. pyogenes* expresses various virulence factors, including but not restricted to neuraminidase H, neuraminidase P, collagen-binding protein A, fimbriae, and pyolysin (PLO), one of the most important factors [2]. Deleting or mutating the plo gene remarkably reduced the pathogenicity of *T. pyogenes* in a mouse model [17]. PLO is a member of the cholesterol-dependent cytolysin (CDC) family, with members including but not restricted to pneumolysin (PLY), listeriolysin O, perfringolysin O, and suilysin [2]. The 3D structures of CDC molecules are similar [2] and are the basis of the four domains’ (D1 to D4) structure of the monomeric PLO molecule. CDCs are expressed as soluble monomers, which adhere to cholesterol-rich membranes by their D4, organize into closed rings as mediated by their D2 and D3, and form a prepore membrane protein complex. This event results in an extensive structural remodeling in which the D3 converses to transmembrane hairpins, the D2 structurally collapses, and the CDC’s prepore complex forms a large oligomeric β-barrel and perforates the plasma membrane [18]. The role of D1 in pore forming remains unclear; however, the replacement of some amino acids impairs the hemolytic activity of PLO molecules [19,20,21]. CDCs can form pores in the cholesterol-containing membrane and are thus further classified as pore-forming toxins (PFTs) [22]. The earliest documented effect of PFTs is their ability to rapidly kill host cells through osmotic lysis; however, recent views suggested that the physiological concentrations of PFTs during bacterial infection are possibly sublytic for the host cells [23]. Sublytic concentrations of PLY can induce the maturation of IL-1β, an important proinflammatory cytokine, in neutrophils, macrophages, and dendritic cells by activating the NLRP3 pathway [24,25]. Therefore, CDCs may show their virulence by causing inflammation. 

The PLO molecule is one of the targets for the development of a *T. pyogenes* vaccine. Early studies treated the supernatant of *T. pyogenes* culture with formalin and then used aluminum hydroxide to absorb the components in the supernatant to synthesize vaccines [6]. However, *T. pyogenes* grows slowly, and animal serum is generally required as a supplement of the culture medium for growth promotion. Thus, this method costs time and money. In addition, the vaccines prepared by this technique generally contain multiple protein components that may lead to biosafety problems or side effects. For example, serum ingredients in the vaccines may spread some infectious diseases, such as mad cow disease, or lead to hypersensitivity reactions in animals. After the identification of the plo gene, some studies used prokaryotic expressed recombinant PLO (rPLO) protein to prepare vaccines [13,17]. However, rPLO is cytotoxic and may cause tissue damage. Detoxification of rPLO with formaldehyde is the solution [17]; however, the process needs fine optimization to ensure effective inactivation without causing the excessive denaturation of antigen molecules and the loss of immunogenicity. Another choice is to prepare vaccines using PLO mutants or truncated PLO molecules with low cytotoxicity. Jost and colleagues reported that the genetic toxoids of PLO provide perfect immunoprotection to mice [10]. Our previous study revealed that the mice that received prokaryote-expressed chimeric antigen, which contains domain 4 of the PLO molecule, were partially protected against *T. pyogenes* challenge [26].

The 1–110 and 190–296 segments of the PLO molecule contain several linear epitopes [27]; hence, we speculated that the recombinant proteins involving the two segments and D4 of PLO may be promising antigens for *T. pyogenes* vaccines. The recombinant antigen molecules may not have the biological functions of the intact PLO molecules and thereby show good safety.

In this study, we constructed two recombinant immunogens named tPLOA1 (involves segments 1–110 and a D4 domain) and tPLOA2 (involves segments 190–296 and a D4 domain). Sheep red blood cell membrane (sRBCM) and incomplete Freund’s adjuvant (IFA) were used as an adjuvant system to prepare vaccines from the two immunogens. The vaccines provided partial immunoprotection for the mice against the lethal challenge of *T. pyogenes*. Our results suggested that the oligomerization and structural change of PLO may lead to the failed immunization of the vaccines based on PLO molecules. We also found that the titer of anti-hemolysis antibodies is not a rational indicator for evaluating the immunoprotective effect of these vaccines.

## 2. Materials and Methods 

### 2.1. Bacterial Strain 

*T. pyogenes* (strain 0912) was isolated from the lung of a bovine with pneumonia (Heilongjiang, China) in our laboratory and cultured in Martin broth medium with 10% fetal bovine serum (FBS) under aerobic conditions [26].

### 2.2. Construction of Recombinant Plasmids 

pET-30a-plo, a recombinant plasmid containing the nucleic acids (designated as mature plo gene in the current study, the 82–1605 bp of the gene encodes an immature PLO molecule) encoding the mature PLO molecule (without the signal peptide) was previously constructed in our laboratory [26]. pET-30a-plo d123, a recombinant plasmid containing the 1–1179 bp of mature plo gene that encodes amino acids 1–393 (1–393aa) of a mature PLO molecule (Domain 1 + Domain 2 + Domain 3 of PLO, D123), was previously constructed in our laboratory [20]. pET-30a-plo d4, a recombinant plasmid containing the 1180–1524 bp of mature plo gene that encodes 394–507aa (Domain 4 of PLO, D4), was previously constructed in our laboratory [26]. Nucleic acids encoding 1–110aa, 190–296aa, and 394–507aa of mature PLO were amplified by polymerase chain reaction (PCR) using the primer pairs listed in Table 1. The genes encoding tPLOA1 and tPLOA2 were obtained using the overlap extension PCR method. Nucleic acids encoding a 10-amino-acid flexible peptide (Gly-Gly-Gly-Gly-Ser-Gly-Gly-Gly-Gly-Gly-Ser) were introduced into tploA1-B-fu, tploA2-B-fu, and plo-d4-F-fu to maintain the conformation of the two parts of the fusion protein. The fused genes were digested using *Eco*R I and *Xho* I and then inserted into a pET-32a (+) plasmid vector. The recombinant plasmids were named as pET-32a-tploA1 and pET-32a-tploA2, respectively. The strategy for constructing the two plasmids is described in detail in Supplementary Figure S1.

### 2.3. Expression and Purification of Recombinant Proteins

pET-30a-plo, pET-30a-plo d123, pET-30a-plo d4, pET-32a-tploA1, and pET-32a-tploA2 were transformed into *Escherichia coli* (*E. coli*) Rosetta (DE3)^TM^ competent cells. Isopropyl-β-D-thiogalactoside (final concentration 0.1 mM) was used to induce the expression of recombinant proteins rPLO, rPLO D123, rPLO D4, tPLOA1, and tPLOA2. The induction of protein expression lasted for 4 h at 37 °C. The expression of proteins was quantified by density analysis via the BandScan v5.0 software (Glyko, Novato, CA, USA) after sodium dodecyl sulfate-polyacrylamide gel electrophoresis (SDS-PAGE). Then, the proteins were purified using nickel-charged resin (binding buffer: 20 mM Na_3_PO_4_, 0.5 M NaCl, 20 mM imidazole, and 8 M urea, pH7.4; washing/eluting buffer: 20 mM Na_3_PO_4_, 0.5 M NaCl, 60–500 mM imidazole, and 8 M urea, pH7.4) and dialyzed against phosphate-buffered saline (PBS) with 5% glycerol at 4 °C for 48 h. The proteins were quantified through a bicinchoninic avid (BCA) method and stored at −80 °C until use. 

### 2.4. Membrane Binding Assay

sRBCM was prepared by suspending 1 mL of prepared sheep red blood cells (sRBCs) in 45 mL of deionized water for 6 h at 4 °C. The mixture was then centrifuged at 4800× *g* for 10 min at 4 °C. The cell membrane pellet was resuspended in 2.5 mL of PBS. tPLOA1 or tPLOA2 was serially diluted into 128, 64, 32, 16, 8, 4, 2, and 1 μg/mL. Then, 200 μL of diluted recombinant proteins were incubated with 200 μL of cell membrane suspension for 30 min at 37 °C. Then, the mixtures were centrifuged at 11,600× *g* for 5 min at 4 °C. Pellets were washed twice with PBS and then resuspended in 15 μL of PBS. The proteins in the samples were denatured in the SDS-PAGE loading buffer and heated in boiled water for 10 min. As controls, rPLO D123 (64 μg/mL) and rPLO D4 (64 μg/mL) were also used to perform the above experiment. However, after the incubation of proteins and sRBCM at 37 °C, the samples were centrifuged at 11,600× *g* for 10 min at 4 °C. The centrifugation pellets were treated as mentioned above, whereas protein components in the centrifugation supernatant were precipitated by the addition of trichloroacetic acid (final concentration is 10%) and then dissolved in 15 μL of 1 M NaOH. The proteins were denatured in the SDS-PAGE loading buffer and heated in boiled water for 10 min. Proteins in the samples were separated using 12% SDS-PAGE and transferred onto a nitrocellulose membrane. Then, Western blot was performed with mouse anti-His tag monoclonal antibodies (TA-02, ZSGB-BIO) (1:1000 diluted) and anti-mouse horseradish peroxidase (HRP)-linked antibodies (ZDR-5307, ZSGB-BIO) (1:5000).

### 2.5. Cytotoxicity Assay of tPLOA1 and tPLOA2

L929 cells, RAW264.7 cells, or Madin-Darby bovine kidney (MDBK) cells were cultured in a Roswell Park Memorial Institute-1640 (RPMI-1640) medium with 10% FBS, seeded in a 96-well cell culture plate at a density of 5 × 10^3^ cells/well, and allowed to grow for 12 h. The culture medium was then discarded, and 100 μL of a fresh culture medium containing tPLOA1 or tPLOA2 was added into each well. Control cells were treated with 100 μL of a fresh culture medium plus PBS. The Cell Counting Kit-8 (CCK8) solution (10 μL) (C0037, Beyotime, Beijing, China) was added into each well. The cells were cultured at 37 °C. The optical density at 450 nm (OD 450) of each well was measured at 30, 60, 90, 120, 240, 360, and 480 min after treatment. Cell viability was shown as the OD 450 value. A well with high OD 450 value contains a high number of live cells.

### 2.6. Animal Immunization and Challenge

Vaccines were prepared either by mixing 100 μL (500 μg/mL) of recombinant proteins with 200 μL of sRBCM suspension and incubating at 37 °C for 30 min or by mixing 100 μL (500 μg/mL) of recombinant proteins with 100 μL of IFA and subjecting to full emulsification.

Eight-week-old clean female Kunming mice (body weight is 18 g to 20 g) were purchased from the Chunyuansu Corp., Harbin, China, provided with food and water ad libitum, and acclimatized to laboratory conditions for 1 week before experimentation. The experimental protocol was approved by the Ethics Committee on the Use and Care of Animals, Northeast Agricultural University, China (2017NEAU-097).

Sixty mice were randomly divided into 10 groups. The mice were immunized three times with different vaccines, namely, PBS, sRBCM, rPLO, or sRBCM-trapped rPLO (Table 2) with an interval of 2 weeks between two adjacent immunizations. Blood samples were drawn on days 7, 21, and 35 from the tail vein, and sera were collected and stored at −80 °C for antibody titer detection. 

Two weeks after the third immunization, the mice were challenged with 2 × 10^9^ colony-forming units (CFU) of *T. pyogenes* (strain 0912) through the intraperitoneal route. The number of surviving mice in each group were recorded every 24 h until 17 days after the challenge. Immunization, specimen collection, and challenge protocol are shown in Supplementary Figure S2.

### 2.7. Enzyme-Linked Immunosorbent Assay (ELISA)

rPLO-specific IgG titers were measured using twofold serial dilutions of serum by indirect ELISA. rPLO (100 ng/well) was coated onto 96-well plates and probed with the collected sera. The plates were then incubated with HRP-conjugated goat anti-mouse IgG (ZDR-5307, ZSGB-BIO, Beijing, China) (1:5000), and absorbance was measured at 450 nm. End-point titers were determined as the maximum antibody dilution at which the OD was twice more than the mean OD of negative sera.

### 2.8. Hemolysis Inhibition Assay 

Purified rPLO was tested for hemolytic activity as described previously [20]. In brief, 25 μL of the twofold serial dilutions of the sera with 1:25 initial dilution ratio was incubated with 4 hemolysis unit of rPLO in a V-bottomed 96-well microtiter plate at 4 °C for 3 h. Subsequently, 50 μL of sRBCs were added to each well, and the mixtures were incubated for another 30 min at 37 °C. The resultant antibody titers were expressed as the reciprocal of the highest dilution times of sera that completely inhibited hemolysis.

### 2.9. Statistical Analysis

Data were presented as means ± standard deviations. One-way or two-way analysis of variance (AVONA) was used for statistical comparisons. Statistical significance was expressed as *p* ≤ 0.05 (*) and *p* ≤ 0.01 (**).

## 3. Results

### 3.1. Prokaryotic Expression and Purification of Recombinant Proteins

**Figure 1:**
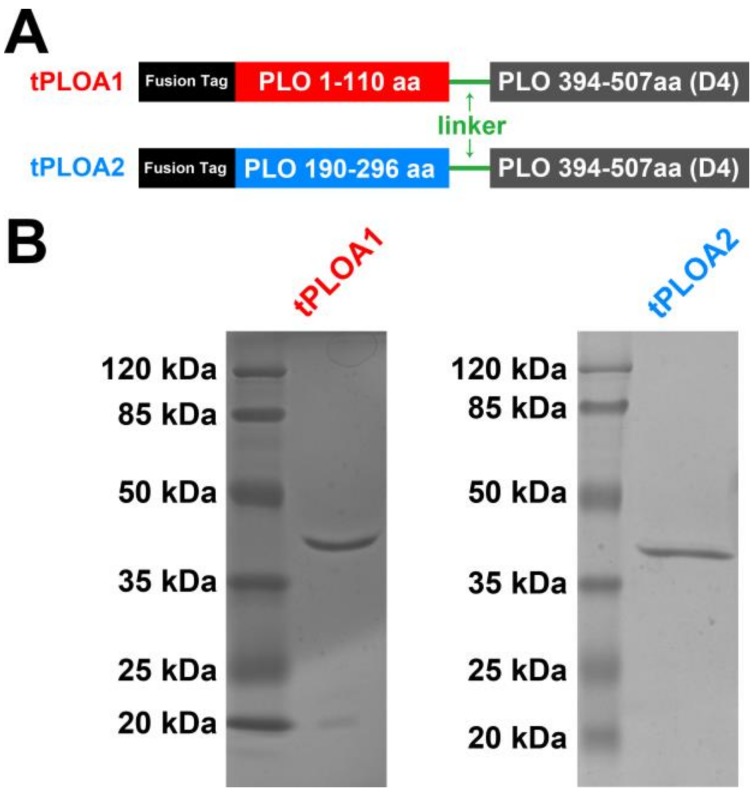
Design and preparation of tPLOA1 and tPLOA2. (**A**) The structure scheme of tPLOA1 and tPLOA2. tPLOA1 contains a segment aa 1–110 (marked red) and a D4 structure (marked gray) of the pyolysin (PLO) molecule. tPLOA2 contains a segment aa 190–296 (marked blue) and a D4 structure (marked gray) of the PLO molecule. Both of the two recombinant proteins contain a polypeptides fusion tag (marked black), which is encoded by pET-32a (+) plasmid, at their amino terminus and a polypeptides linker (marked green) between the amino terminus part and the D4 structure of the recombinant proteins; (**B**) SDS-PAGE results showed the purified tPLOA1 and tPLOA2. The two recombinant proteins showed similar molecular weight.

Figure 1A shows the scheme of the structures of tPLOA1 and tPLOA2. The expression level was approximately 40% of the total bacterial protein. tPLOA1 and tPLOA2 were purified by affinity chromatography. The molecular weight of tPLOA1 and tPLOA2 was approximately 40 kDa according to SDS-PAGE and is in accordance with the predicted value (Figure 1B). The concentration of purified recombinant proteins was approximately 400 mg/mL.

### 3.2. Determination of the Cytotoxicity of tPLOA1 and tPLOA2

**Figure 2:**
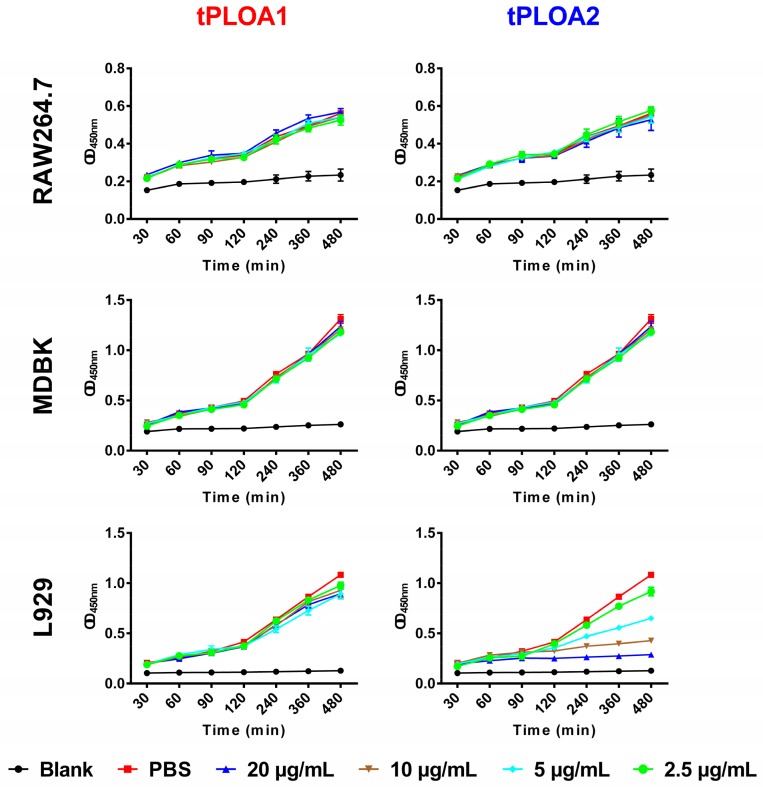
Cytotoxicity of tPLOA1 and tPLOA2 to different cells. tPLOA1 showed no cytotoxicity to RAW264.7, MDBK, and L929 cells. tPLOA2 showed no cytotoxicity to RAW264.7 cells. In MDBK cells, 20 μg/mL tPLOA2 showed slight cytotoxicity, while, in L929 cells, tPLO2 showed cytotoxicity at concentrations higher than 5 μg/mL. The assay was technically repeated for three times.

MDBK, RAW264.7, and L929 cells were treated with different concentrations of the two proteins to determine their cytotoxicity. CCK8 solution was also added in each well to monitor cell viability. CCK8 assays showed that the tPLOA1 treatment did not decrease the viability of MDBK, RAW264.7, and L929 cells within 6 h even at a concentration as high as 20 μg/mL (Figure 2). tPLOA2 (20 μg/mL) also showed no cytotoxicity on RAW264.7 cells but decreased the viability of MDBK cells after 4 h. Compared with MDBK cells, L929 cells were more sensitive to tPLOA2, and their viability was decreased by tPLOA2 at concentrations higher than 5 μg/mL (Figure 2).

### 3.3. Determination of the Binding Capacity of the Two Recombinant Proteins to sRBCM

**Figure 3:**
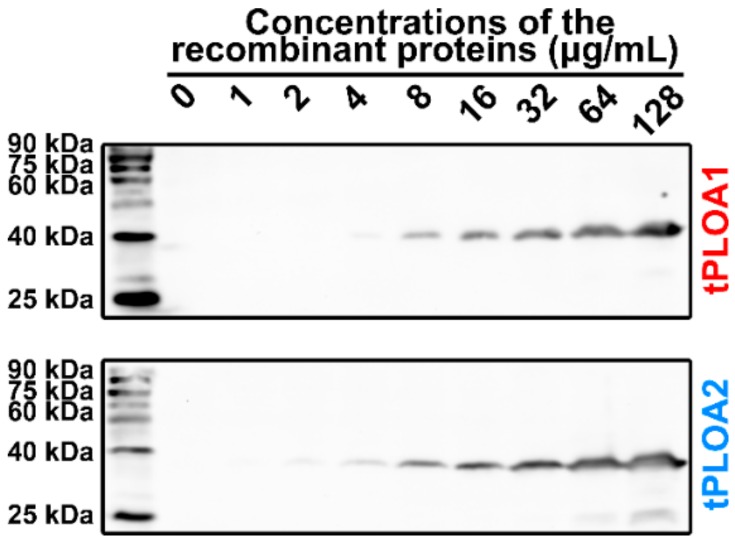
Results of cell membrane binding assays. Both tPLOA1 and tPLOA2 could bind to sRBCM. The results indicated that the D4 structures in tPLOA1 and tPLOA2 keep the cholosterol-binding function. Thus, in the following experiment, sheep red blood cell membrane (sRBCM) could be used as an adjuvant for preparing vaccines. This assay was performed for at least three times.

tPLOA1 and tPLOA2 were incubated with sRBCM for 30 min to determine the cell membrane binding capacity of the two proteins. As shown in Figure 3, tPLOA1 and tPLOA2 could bind to sRBCM. Many tPLOA1 or tPLOA2 molecules were trapped by sRBCM when the concentration of the two recombinant proteins in the mixture was increased. Binding assay was also performed by using rPLO D123 and rPLO D4 to determine whether the binding of these two proteins to sRBCM can be attributed to the unspecific adsorption of sRBCM to the proteins. The results confirmed the binding to sRBCM by rPLO D4 but not by rPLO D123 (Supplementary Figure S3). This finding indicated that proteins with the D4 structure of a PLO molecule could bind to sRBCM. Thus, the binding of tPLOA1 and tPLOA2 was not attributed to the unspecific adsorption of sRBCM to the proteins.

### 3.4. Titration of the PLO-Specific IgG in Mice Sera by ELISA

**Figure 4:**
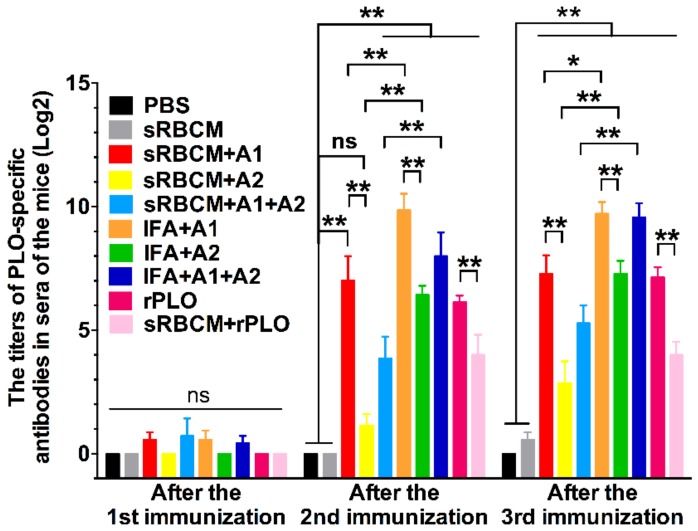
Titration of rPLO-specific IgG in sera of immunized mice by ELISA. sRBCM-trapped recombinant proteins were not as potent as incomplete Freund’s adjuvant (IFA) emulsified recombinant proteins in inducing rPLO-specific IgG. Compared with tPLOA2 (A2), tPLOA1 (A1) was more potent in eliciting rPLO-specific IgG in mice. Binding to sRBCM significantly reduced the potency of rPLO in eliciting rPLO-specific IgG. (* *p* < 0.05; ** *p* < 0.05; ns: not significant).

ELISA was performed on the sera of 60 mice to determine the levels of their PLO-specific IgG. The results showed that all the vaccines could elicit PLO-specific IgG in mice (Figure 4). After the second immunization, the immunized mice, except for those administered with sRBCM-trapped tPLOA2, produced a remarkably higher level of rPLO-specific IgG than the PBS and sRBCM-inoculated groups. After the third immunization, the mice in all vaccinated groups showed a substantially higher level of rPLO-specific IgG than those in the PBS and sRBCM-inoculated groups. The sera from mice that received three doses of sRBCM reacted slightly with rPLO in ELISA. However, no statistical difference was found in the rPLO-specific IgG levels between the sera from the mice that received sRBCM and those from the PBS group. 

IFA-emulsified recombinant proteins elicited a considerably higher level of PLO-specific IgG than the corresponding recombinant proteins trapped by sRBCM in mice (Figure 4). The mice immunized with sRBMC-trapped rPLO showed a remarkably lower level of PLO-specific IgG than those that received rPLO without any adjuvant (Figure 4).

rPLOA1 is more potent in eliciting PLO-specific IgG than rPLOA2. rPLOA1-immunized mice showed a higher level of PLO-specific IgG than rPLOA2-immunized mice regardless of using sRBCM or IFA as the adjuvant system (Figure 4).

The PLO-specific IgG level of mice that received IFA-emulsified tPLOA1 + tPLOA2 was similar to that of mice that received IFA-emulsified tPLOA1 after the third immunization (Figure 4). The mice that received sRBCM-trapped tPLOA1 + tPLOA2 showed a substantially lower level of PLO-specific IgG than those administered with sRBCM-trapped tPLOA1 (Figure 4).

### 3.5. Determination of the Titers of Anti-Hemolysis Antibodies in Mice Sera

**Figure 5:**
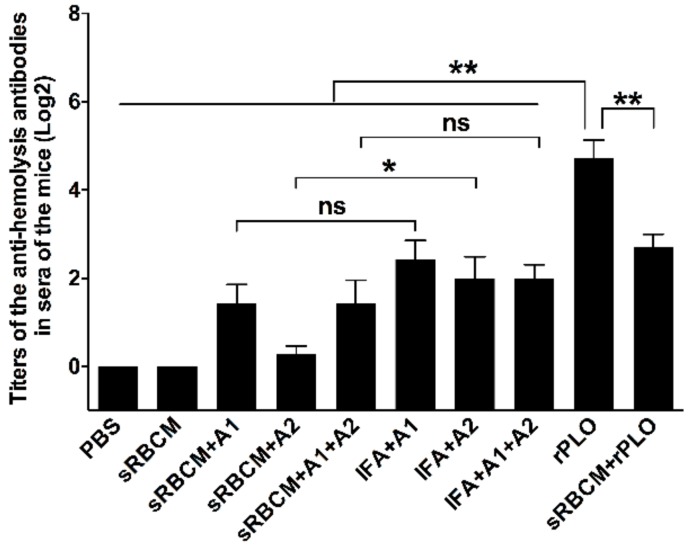
Titers of anti-hemolysis antibody in sera of immunized mice. Sera of rPLO immunized mice showed the highest level of anti-hemolysis antibody in all sera. Compared with the sera that were collected from mice received sRBCM-trapped tPLOA2 (A2), sera from mice received IFA emulsified tPLOA2 showed a significantly higher level of anti-hemolysis antibody. One-way ANOVA test was performed. (* *p* < 0.05; ** *p* < 0.05; ns: not significant).

The sera collected from all 60 mice after the third immunization were also used in the anti-hemolysis assay. The sera from unimmunized mice and those administered with sRBCM showed no anti-hemolysis effect in the rPLO-sRBC system. The sera from rPLO-immunized mice showed substantially higher anti-hemolysis activity compared with those from other mice (Figure 5). The sera from mice immunized with sRBCM-trapped tPLOA2 showed remarkably lower anti-hemolysis activity than those from the mice immunized with IFA-emulsified tPLOA1, tPLOA2, and tPLOA1 + tPLOA2 and sRBCM-trapped rPLO.

### 3.6. Bacterial Challenge

The mice were challenged with 2 × 10^9^ CFU *T. pyogenes* two weeks after the third immunization. All mice showed messy hair, sluggishness, and anorexia within 12 h after the bacterial challenge. Some of the mice also exhibited nervous system disorders, such as twitching, before death. Most of the surviving mice recovered within seven days after the challenge.

**Figure 6:**
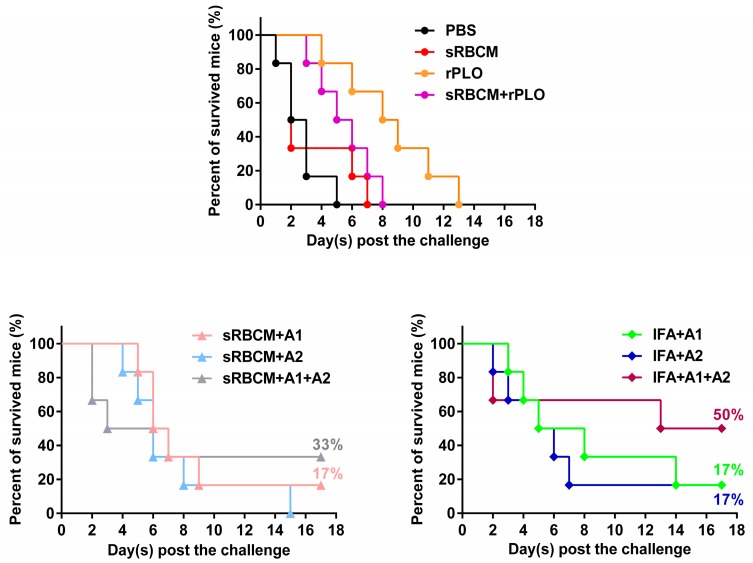
Survival curve of mice after the lethal challenge with *Trueperella pyogenes* (*T. pyogenes*). A total of 50% of the mice that received the IFA emulsified tPLOA1 (A1) + tPLOA2 (A2) survived the challenge. Moreover, 33% of the mice that received the sRBCM-trapped tPLOA1 + tPLOA2 survived the challenge. Furthermore, 17% of the mice that received IFA emulsified tPLOA1 or tPLOA2 and the mice that received sRBCM-trapped tPLOA1 survived the challenge. The mice in other groups died within 15 days after the challenge.

As shown in Figure 6, the mice in the PBS group and those administered with sRBCM, rPLO, sRBCM-trapped rPLO, and sRBCM-trapped tPLOA2 died within 15 days after the challenge. Survival after challenge was approximately 17% for the mice that received IFA-emulsified tPLOA1 or tPLOA2 and sRBCM-trapped tPLOA1, 33% for the mice immunized with sRBCM-trapped tPLOA1 + tPLOA2, and 50% for the mice that received IFA-emulsified tPLOA1 + tPLOA2.

The mice in the PBS group died within five days, and those administered with sRBCM died within seven days (Figure 6). Compared with the mice that received PBS or sRBCM, those in the immunized groups showed different levels of resistance to the challenge. The death of mice in PBS and sRBCM groups occurred within 24 h after the challenge. The death of immunized mice was postponed because no immunized mouse died within the first 24 h after the challenge.

The mice that received sRBCM-trapped rPLO died within eight days, and three out of the six mice that received rPLO survived longer than eight days after the challenge (Figure 6) but died within 13 days (Figure 6).

## 4. Discussion

Although *T. pyogenes* is an economically important pathogen, only one *T. pyogenes* vaccine is commercially available. Therefore, the development of effective vaccines against *T. pyogenes* infections is still necessary.

In this study, we designed and prepared two recombinant antigens (tPLOA1 and tPLOA2) that are involved in the immunodominant areas of the D123 of PLO molecules. Both recombinant proteins have only two-fifths of the primary structure of a PLO molecule and thus showed no or slight cytotoxicity in cultured cells (Figure 2). In the animal experiment, the mice that received two recombinant antigens did not show any discomfort, and no lesion of the skin around the injection sites was observed. Thus, the recombinant antigens exhibit good safety.

Although the recombinant antigens are safe for animals, the immune challenge experiment showed that the vaccines could only protect up to 50% of the mice. This result suggests that the antigens and vaccine formulations are not sufficiently potent. Nevertheless, some observed phenomena are interesting and maybe valuable for the development of new *T. pyogenes* vaccines.

The structural change and oligomerization of PLO molecules may affect their potency in inducing protective antibodies. According to the results, sRBCM-trapped rPLO was less capable of inducing PLO-specific IgG in mice than rPLO (Figure 4). As a member of the CDC family, PLO can bind and form pores on cholesterol-containing membranes. In pore formation, CDC monomers assemble into large oligomers and undergo an extensive structural change [18]. This oligomerization and structural change might have led to the loss of some conformational epitopes of the PLO monomer molecule and the decrease of accessibility of some linear epitopes in PLO monomers. This event may explain the low titer of PLO-specific IgG in the mice that received sRBCM-trapped rPLO.

Another piece of evidence that supports the above speculation is that the level of PLO-specific IgG of mice that received sRBCM-trapped tPLOA2 was substantially lower than that of mice that received sRBCM-trapped tPLOA1 (Figure 4). Amino acid segments 1–110 and 190–296 are located at the D2 and D3 of the PLO molecule, respectively [27]. The D3 of CDC molecules forms the inner side of membrane-penetrating pores after an entirely structural refolding, and the D2 of CDC molecules undergoes a simple rotation and forms the outer side of the pores when the prepore complexes transform to pores [28]. Therefore, the accessibility of the epitopes in D3 is minimal compared with that in D2 after the oligomerization and conformational change of PLO molecules. According to the results in Figure 2, tPLOA2 at concentrations higher than 5 μg/mL showed cytotoxicity in L929 cells. However, tPLOA1, which also has a D4 structure, showed no cytotoxicity in L929 cells even at a concentration as high as 20 μg/mL. Hence, the cytotoxicity of tPLOA2 to L929 cells should be attributed to segments 190–296. This result indicated that the 190–296 fragment of the PLO molecule preserved the structural rearrangement and cell membrane insertion ability to some extent. The conformational change of segments 190–296 might decrease the accessibility of its epitopes. The difference in the titers of the rPLO-specific IgG of mice that received sRBCM-trapped tPLOA1 or tPLOA2 is mainly attributed to the variations in the immunogenicity of segments 1–110 and 190–296. IFA-emulsified tPLOA1 elicited a higher level of rPLO-specific IgG in mice compared with IFA-emulsified tPLOA2; hence, the immunogenicity of tPLOA1 is better than that of tPLOA2 (Figure 4). However, the rPLO-specific IgG titer of the mice that received IFA-emulsified tPLOA2 was approximately two-thirds and three-fourths of the titer of the mice that received IFA-emulsified tPLOA1 after the second and third immunizations, respectively. When sRBCM was used as adjuvant, the rPLO-specific IgG titer of mice that received tPLOA2 was only one-third and one-half of the titer of the mice that received tPLOA1 after the second and third immunizations, respectively (Figure 4). tPLOA2 molecules lose more IgG-eliciting potency than tPLOA1 molecules after binding with sRBCM. Thus, the structural change of the D3 domain is an important factor that affects the antibody-inducing potency of the PLO molecules.

Our results and the speculation mentioned above indicate that sRBCM is not a rational adjuvant system for the synthesized antigens. In a previous study, Hu and colleagues used mouse RBCM-coated poly (lactic-co-glycolic acid) nanoparticles as an adjuvant system of staphylococcal α-hemolysin (Hla) (another well-known PFT) based vaccine and obtained promising results, which indicated that nanoparticle-detained toxin elicited higher levels of anti-Hla IgG titers and showed superior protective immunity against toxin-mediated adverse effects in a mouse model compared with heat-inactivated Hla [29]. The discrepancy between our results and Hu’s results might be attributed to the different conformational change patterns of CDCs and Hla when they form pores in the cell membrane. The structural changes of Hla molecules during pore formation [30] are not as extensive as those of CDCs [28]; therefore, Hla might preserve more naïve structural characteristics and epitopes than CDCs in the formed pore complex. 

Although tPLOA2 was not as potent as tPLOA1 in eliciting PLO-specific IgG, its involvement improved the performance of the vaccines. The mice that received mixtures of tPLOA1 and tPLOA2 showed a higher survival rate after challenge compared with the mice that received either of the two proteins (Figure 6). tPLOA1 and tPLOA2 have similar molecular weight; hence, 50 μg of either of the two proteins possesses an equal amount of D4 segments to the mixture of 25 μg tPLOA1 plus 25 μg tPLOA2. This finding indicates that the better performance of the mixture of tPLOA1 and tPLOA2 in eliciting immunoprotection is not attributed to the D4 segment but the existence of segments 1–110 and 190–296. Thus, both areas of the PLO molecule are essential in eliciting immunoprotection in mice. We also constructed an oligonucleotide that encodes a chimeric protein involving segments 1–110, 190–296, and D4 of PLO. However, the chimeric protein failed to be expressed in the *E. coli* expression system. No observable expression could be detected by SDS-PAGE. We speculated that the structure of the mRNA is extremely complex and hindered the transcription of the protein.

Another observation is that the titer of the anti-hemolysis antibodies is not a rational indicator for predicting the immunoprotective effect of PLO-based vaccines against *T. pyogenes* challenge. rPLO, even without any adjuvant, elicited a remarkably higher level of anti-hemolysis antibodies than IFA-emulsified recombinant antigens after immunization (Figure 5). However, rPLO-immunized mice died within 13 days after the challenge, whereas 50% of the mice that received IFA-emulsified tPLOA1 + tPLOA2 and 30% of the mice that received RBMC-trapped tPLOA1 + tPLOA2 survived the lethal challenge (Figure 6). Our result is consistent with a previous study, which showed that high-level anti-hemolysis antibodies are elicited by alum-precipitated PLO toxoids but fail to protect cattle from summer mastitis [6]. The authors suggested that the antitoxin might not be the antibodies important for protection.

From our perspective, PLO-induced anti-hemolysis antibodies did not provide good protection to mice because the oligomerization and structural changes of the PLO molecules hindered the binding of the antibodies. The molecular weight of tPLOA1 and tPLOA2 is approximately two-fifths of the molecular weight of a PLO molecule, and 50 μg of tPLOA1 or tPLOA2 possesses more D4 domain than 50 μg of rPLO. Meanwhile, IFA was used to improve the performance of tPLOA1 and tPLOA2. Thus, IFA-emulsified tPLOA1 or tPLOA2 should elicit more D4-specific antibodies compared with rPLO. If the anti-hemolysis effect of the sera is mainly attributed to the existence of D4-specific antibodies, then the sera from the mice that received IFA-emulsified tPLOA1 or tPLOA2 should perform better than sera from the mice that received rPLO in the anti-hemolysis assay. However, our research showed an opposite result. Thus, the antibodies targeting the D123 of the PLO molecule contributed substantially to the anti-hemolysis activity of the sera from the mice that received rPLO. In anti-hemolysis assay, rPLO was initially incubated with the antisera before sRBCs were added. In vivo, the PLO molecules had many chances to bind to the cell membrane and form pores before its interaction with the specific antibodies. The anti-hemolysis antibodies failed to bind with PLO due the oligomerization of these molecules and the structural change of D123 and therefore lost their protective effect. This phenomenon may explain why a high level of anti-hemolysis antibodies did not confer good immunoprotection to mice. 

The number of surviving tPLOA1- and tPLOA2-immunized mice was higher than that of rPLO-immunized mice. One of the reasons might be that the antibodies induced by some of the linear epitopes in segments 1–110 and 190–296 could bind with PLO molecules even after the oligomerization and structural change of the molecules. These antibodies could help the immune system to rapidly kill cells with PLO pore complexes by mediating the antibody-dependent cell-mediated cytotoxicity effect. CDCs can activate various intracellular events and thereby increase the expression of proinflammatory cytokines rather than rapidly kill the target cells [24,25]. Thus, the rapid killing of these impaired cells by the host’s immune system would benefit the host by alleviating the inflammation caused by CDCs.

## 5. Conclusions

tPLOA1 and tPLOA2 inoculation can partially protect mice against *T. pyogenes* challenge. We suggest that cell membrane binding, oligomerization, and structural change might be the reasons for the failure of PLO-based vaccines in providing good immunoprotection. A new strategy for developing *T. pyogenes* vaccines is still needed.

## Figures and Tables

**Table 1:** Sequence of the PCR primers used in this study.

Name	Sequence	Restriction Endonuclease Cleavage Sites	Length of the PCR Products
tploA1-F-fu	5′-CCGGAATTCGGATTGGGAAACAGC-3′	*Eco*R Ι	369 bp
tploA1-B-fu	5′-CGAGCCGCCACCACCAGACCCACCACCGCCTAACACGAGCGCGCCAGGAT-3′		
tploA2-F-fu	5′- CCGGAATTCTCAAAGCGTCAACTG-3′	*Eco*R Ι	360 bp
tploA2-B-fu	5′-CGAGCCGCCACCACCAGACCCACCACCGCCAAAAGCCGCTTGTACATCAT-3′	--	
plo-d4-F-fu ^a^	5′-GGCGGTGGTGGGTCTGGTGGTGGCGGCTCGACTTACAAGTCTGGTGAGATC-3′	--	384 bp
plo-d4-B-fu ^a^	5′-CCGCTCGAGCTAGGATTTGACATTGTCCTC-3′	*Xho* I	

The sequence corresponding to Glycine linker was given in lowercase. The sequence corresponding to restriction endonuclease cleavage sites were given underlined. ^a^ The primers were reported in one of our previous studies [26].

**Table 2:** Preparations used to immunize mice.

Number of Mice	Preparations	Composition
6	PBS	300 μL PBS
6	sRBCM	200 μL sRBCM suspension + 100 μL PBS
6	sRBCM + A1	sRBCM + 50 μg tPLOA1
6	sRBCM + A2	sRBCM + 50 μg tPLOA2
6	sRBCM + A1 + A2	sRBCM + 25 μg tPLOA1 + 25 μg tPLOA2
6	IFA + A1	IFA + 50 μg tPLOA1
6	IFA + A2	IFA + 50 μg tPLOA2
6	IFA + A1 + A2	IFA + 25 μg tPLOA1 + 25 μg tPLOA2
6	rPLO	50 μg rPLO
6	sRBCM + rPLO	sRBCM + 50 μg rPLO

## Supplementary Materials

The following are available online at https://www.mdpi.com/2076-393X/8/1/79/s1, Figure S1: Strategy for the construction of recombinant expression plasmids, Figure S2: Scheme of animal immunization and challenge experiment, Figure S3: Determination of the cell membrane binding capacity of rPLO D123 and rPLO D4.
