# Supplementary material for: Evaluation of the Potency of Two Pyolysin-Derived Recombinant Proteins as Vaccine Candidates of Trueperella Pyogenes in a Mouse Model: Pyolysin Oligomerization and Structural Change Affect the Efficacy of Pyolysin-Based Vaccines
Source: Vaccines (Basel). 2020 Feb 10;8(1):79. doi: 10.3390/vaccines8010079 (PMC7157609; doi:10.3390/vaccines8010079)
Supplement: Supplementary file 1 [file vaccines-08-00079-s001.pdf]

vaccines

MDPI

Communication

Evaluation of the Potency of Two Pyolysin-Derived Recombinant Proteins as Vaccine Candidates of *Trueperella Pyogenes* in a Mouse Model: Pyolysin Oligomerization and Structural Change Affect the Efficacy of Pyolysin-Based Vaccines

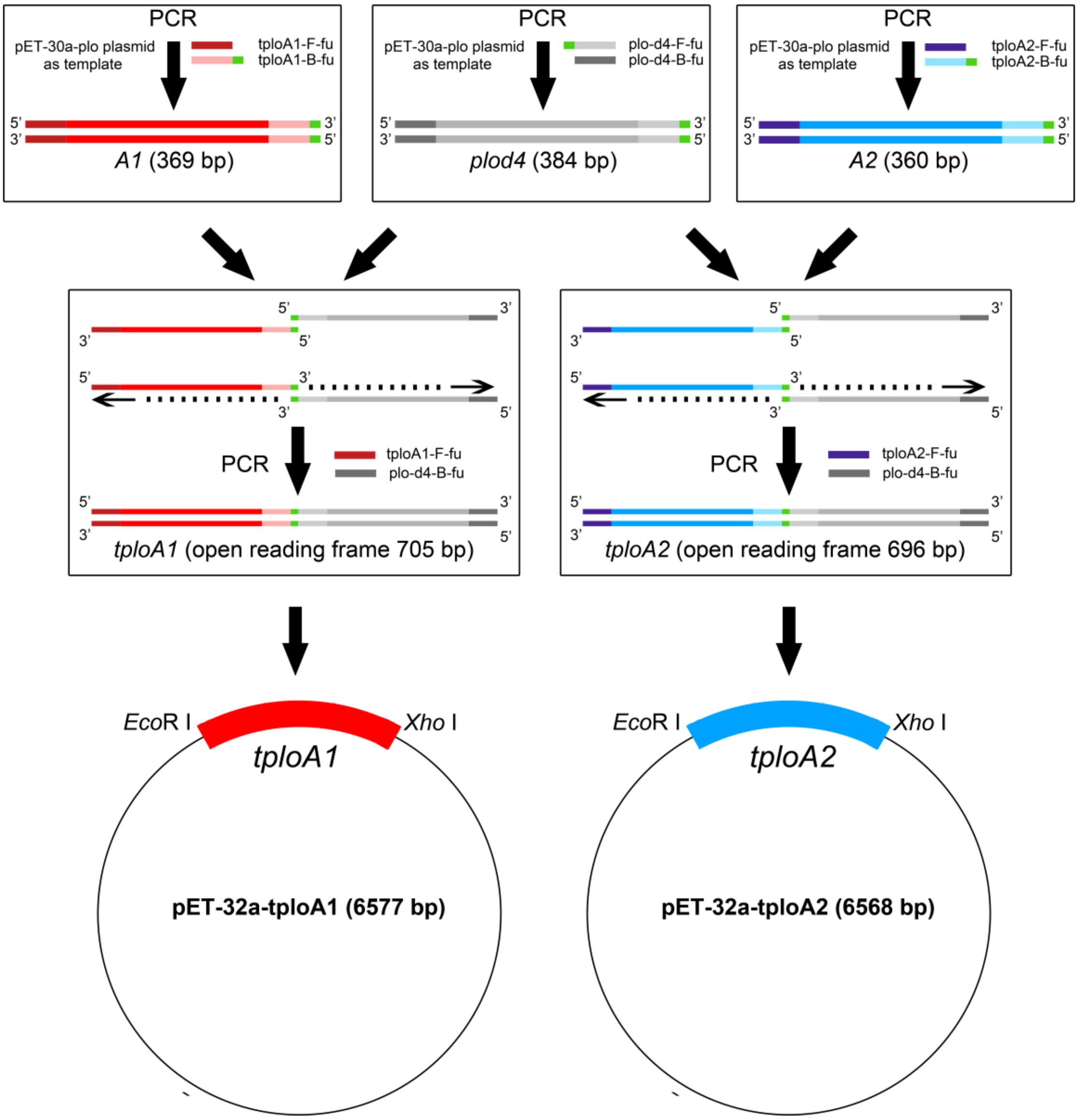

Supplemental Figure S1. Strategy for the construction of recombinant expression plasmids.

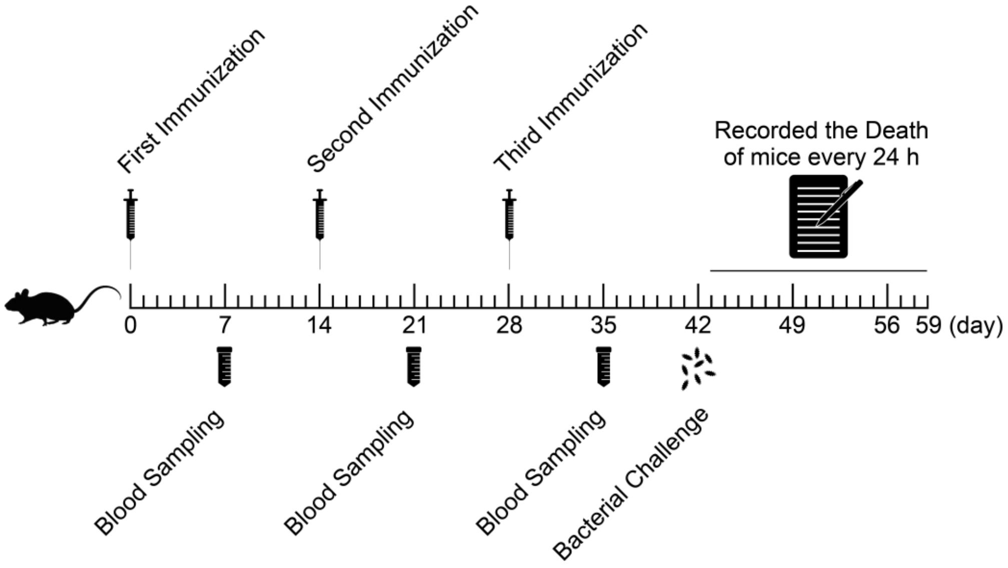

Supplemental Figure S2. Scheme of animal immunization and challenge experiment.

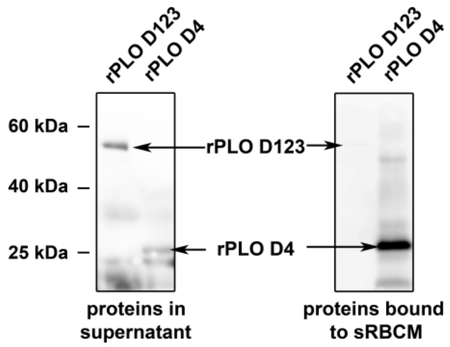

Supplemental Figure 3. Determination of the cell membrane binding capacity of rPLO D123 and rPLO D4. After the incubation with sRBCM, almost all of the rPLO D123 remained in the supernatant, while, only a little rPLO D4 remained in the supernatant (left panel). In contrast, in the sRBCM pellet fraction, only an extremely weak band that represents the rPLO D123 was observed on the western blot result, while, a large amount of rPLO D4 could be detected (right panel). The results indicated that rPLO D4, but not rPLO D123, could bind to sRBCM.

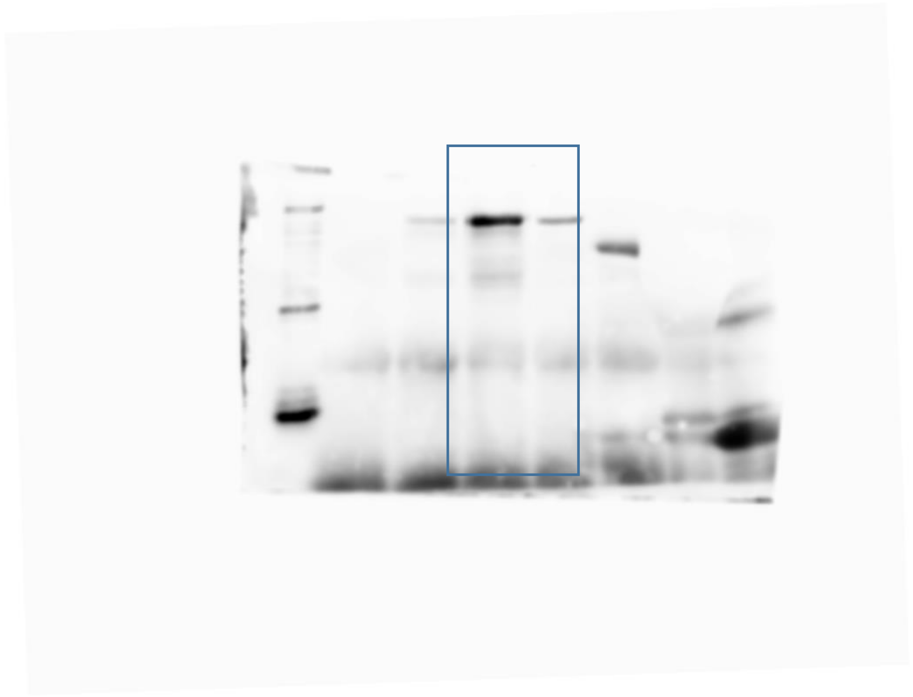

Supplemental data-original western blot pictures. Left panel of supplemental Figure S3.

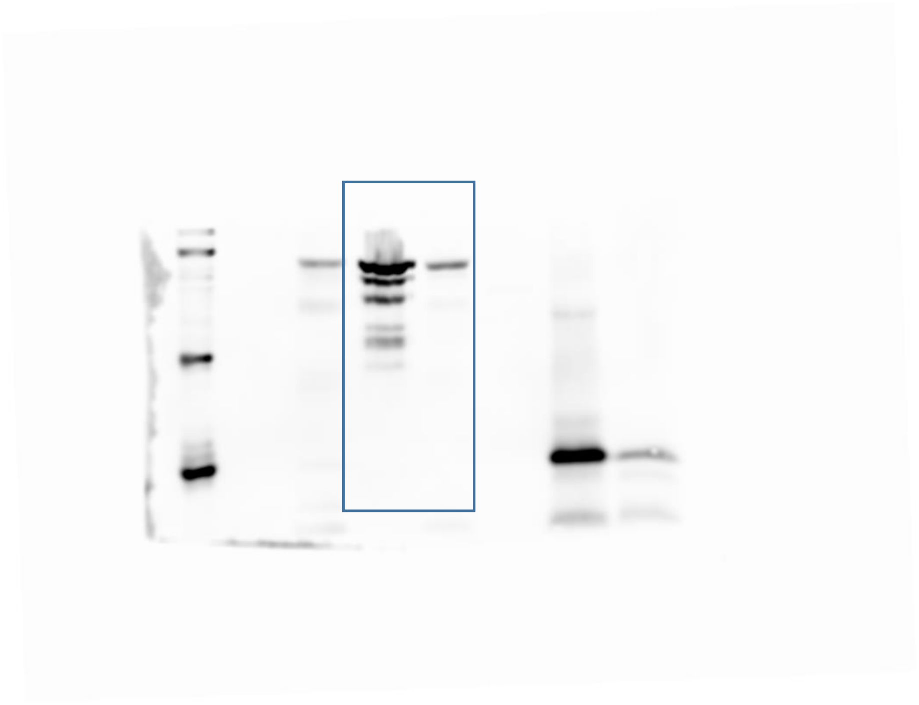

Supplemental data-original western blot pictures. Right panel of supplemental Figure S3.

© 2020 by the authors. Submitted for possible open access publication under the terms and conditions of the Creative Commons Attribution (CC BY) license (http://creativecommons.org/licenses/by/4.0/).
